# Dr. W. H. Park

**Published:** 1885-12

**Authors:** 


### NECROLOGY.

### DEATH OF DR. W. H. PARK, OF TYLER, TEXAS.

Another WARRIOR has fallen! In full armor,—in the midst of pestilence,—battling with "that invisible foe that walketh in darkness," he fell! In the prime and pride of matured manhood he is cut off;—and a gap in the ranks of medicine is made, and in the social circle a void, which will fill many hearts with desolation.

On November 12, we received a note from Dr. S. F. Starley, conveying the sad news of the death of the distinguished Surgeon and Physician, Dr. WILLIAM HENRY PARK, of Tyler. He died on the 4th November, of dengue and the nervous prostration which follows it, increased, no doubt, by heavy and ceaseless labor during the epidemic;—for, an enthusiast in medicine, and a philanthropist, so long as duty called, he was insensible to fatigue—absolutely regardless of self!

Dr. Park was born in Lowndesboro, Ala., January 15, 1836, and was in the 49th year of his age. He was a graduate of the Medical Department of the University of New York, of the class of 1858, and took an *ad eundem* degree at Bellevue in 1872. He was also an A. M. of the Literary College of Alabama. Dr. Park served with distinction as a Confederate Surgeon during the war, and for a number of years was, and up to his death, President of the Board of Medical Examiners for his District, and Surgeon of the I. & G. N. R. R. system. He was a permanent member of the American Medical Association, and of the Texas State Medical Association, and was also a member of the Physicians' Mutual Benefit Association of Texas. He leaves a wife, but no children.

Peace to his ashes!

